# Exophytic colonic carcinoma complicated by gastrocolic fistula mimicking a giant gastric mass: a case report and brief review

**DOI:** 10.3389/fonc.2025.1655501

**Published:** 2025-11-07

**Authors:** Xiaofeng Deng, Zequn Zhang

**Affiliations:** Department of General Surgery, The Second Xiangya Hospital of Central South University, Changsha, China

**Keywords:** exophytic colon cancer, gastric tumor, diagnostic error, gastrocolicfistula, en bloc resection

## Abstract

Colon cancer rarely presents as a giant exophytic mass that mimics a primary gastric malignancy. Such misleading features can result in diagnostic pitfalls and inappropriate surgical planning. We herein report a unique case of colonic adenocarcinoma involving the gastric wall and forming a gastrocolic fistula, initially misdiagnosed as gastric cancer. Moreover, we conducted a brief literature review on the diagnostic challenges associated with exophytic colonic carcinomas to enhance the understanding and management of this rare entity. A 60-year-old man presented with a one-month history of melena and fatigue. Initial abdominal CT and gastroscopy at a local hospital revealed a large exophytic mass occupying the gastric body, leading to a provisional diagnosis of gastric cancer. Biopsy suggested high-grade intraepithelial neoplasia with focal carcinoma. At our center, further evaluation including colonoscopy and PET/CT revealed a mass at the splenic flexure of the colon infiltrating the stomach wall. A multidisciplinary team discussion raised suspicion for colonic origin. The patient underwent en bloc resection including extended left hemicolectomy, partial gastrectomy, lymphadenectomy, distal pancreatectomy, splenectomy and postoperative HIPEC. Surgical findings confirmed a gastrocolic fistula. Final pathology revealed moderately-poorly differentiated adenocarcinoma of colonic origin invading the gastric wall. The patient recovered well postoperatively and completed six cycles of adjuvant chemotherapy, remaining recurrence-free at the 12-month follow-up. This case highlights a rare presentation of splenic flexure colon cancer mimicking a gastric tumor, underlining the importance of thorough preoperative evaluation, multidisciplinary discussion, and awareness of diagnostic traps in trans-organ gastrointestinal oncology.

## Introduction

Colon cancer is a common malignancy worldwide and typically presents with symptoms such as altered bowel habits, rectal bleeding, and abdominal pain (1). However, transverse colon cancers may remain clinically silent until they reach an advanced stage, occasionally presenting with non-specific or misleading symptoms (2, 3). In exceedingly rare instances, colonic tumors can mimic primary gastric malignancies, particularly when they exhibit exophytic growth patterns and invade adjacent organs such as the stomach, gallbladder and ovaries etc (4). These unusual presentations pose significant diagnostic challenges and may lead to misdirected initial management. Here, we report a rare case of exophytic splenic flexure cancer presenting as a giant exophytic mass suggestive of a primary gastric tumor, ultimately found to be a colon cancer with direct gastric invasion and formation of a gastrocolic fistula. This paper deepens the collective understanding of trans-organ invasion patterns of gastrointestinal cancer.

## Case report

**Figure 1:**
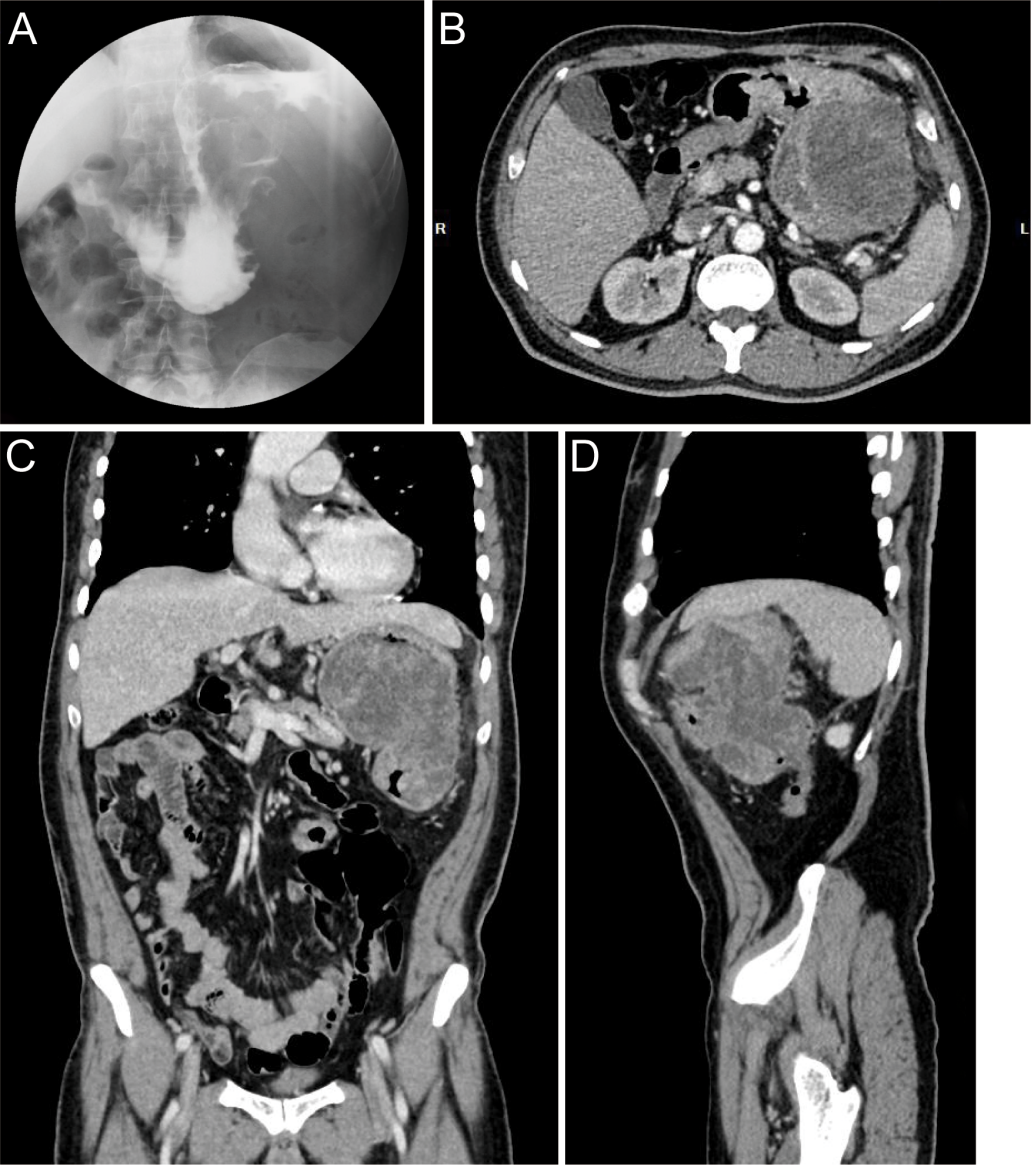
Preoperative radiographic images. **(A)** Upper GI series with barium contrast showing a large filling defect in the greater curvature gastric wall. Axial **(B)**, coronal **(C)**, and sagittal **(D)** contrast-enhanced CT images documenting a large mass contiguous with the gastric body.

**Figure 2:**
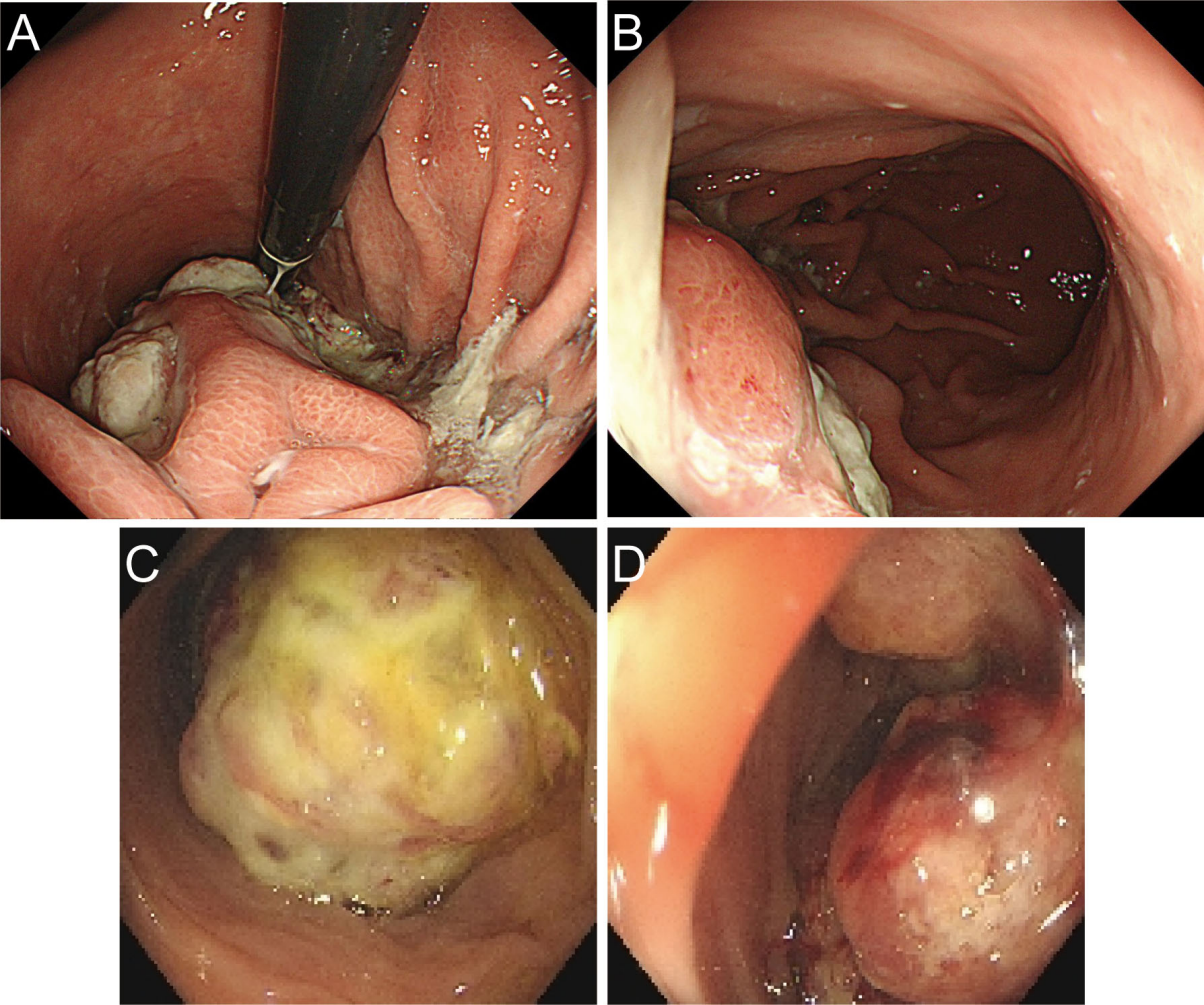
Preoperative endoscopic images. **(A, B)** Gastroscopic images demonstrating a giant irregular protrusion along the greater curvature of the gastric body. **(C, D)** Colonoscopic views revealing an ulcerated mass at the splenic flexure with significant luminal narrowing.

A 60-year-old male presented with a 30-day history of melena and progressive fatigue without hematemesis, nausea, or abdominal distension. Physical examination revealed marked pallor, and laboratory testing confirmed severe anemia (hemoglobin 64 g/L). Initial contrast-enhanced abdominal CT at a local hospital demonstrated a huge hypodense intragastric mass. Following referral to a tertiary center, repeat CT identified an exophytic gastric lesion with multiple perigastric lymph nodes (**Figure 1**). Upper endoscopy revealed a giant irregular protrusion occupying the entire gastric body along the greater curvature (**Figures 2A, B**). Biopsies showed high-grade intraepithelial neoplasia with focal carcinomatous transformation, with Helicobacter pylori immunohistochemistry (IHC) negative. A provisional diagnosis of gastric adenocarcinoma was established, and radical gastrectomy was planned.

**Figure 3:**
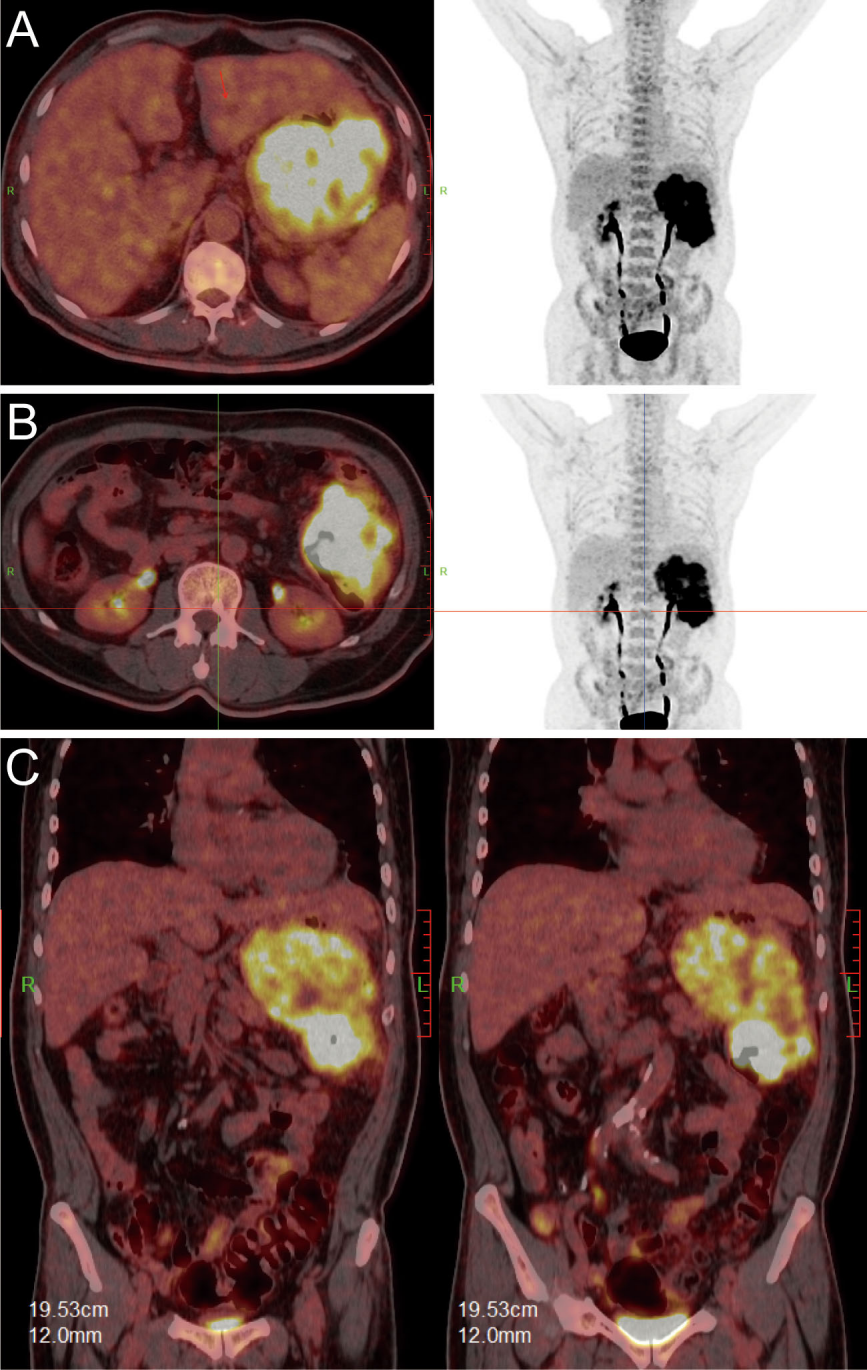
PET/CT fusion images. [**(A, B)**: axial; **(C)**: coronal] demonstrating hypermetabolic tumor involvement of the splenic flexure (SUVmax 39).

Upon transfer to our institution, multidisciplinary review noted an inconsistency: the exophytic morphology contradicted typical gastric cancer phenotypes. Moreover, the possibility of lower gastrointestinal (GI) bleeding could not be entirely ruled out. PET/CT demonstrated intense FDG avidity (SUVmax 39) in a mass bridging the gastric body and splenic flexure, with infiltration of pericolonic fat and metabolically active lymph nodes (**Figure 3**). Subsequent colonoscopy uncovered a protruded mass projecting into the lumen with an eroded surface at the splenic flexure (**Figures 2C, D**), prompting revision of the diagnosis to primary colonic malignancy with gastric invasion. What warrants particular attention and differentiation here is that, unlike typical gastric cancers characterized by ulcerative or infiltrative growth patterns originating from the mucosal epithelium with early lymphatic spread, exophytic gastric tumors demonstrate expansive growth and pathological heterogeneity. These masses may arise from submucosal tissues (e.g., GIST) or represent direct invasion from adjacent organs (e.g., colon cancer). Their intact overlying mucosa often leads to false-negative conventional biopsies, creating a diagnostic pitfall. Furthermore, their distinct biological behavior (predominantly hematogenous metastasis) and therapeutic requirements (either organ-sparing resection or multivisceral resection) necessitate precise origin determination through advanced imaging and deep-tissue sampling before intervention.

**Figure 4:**
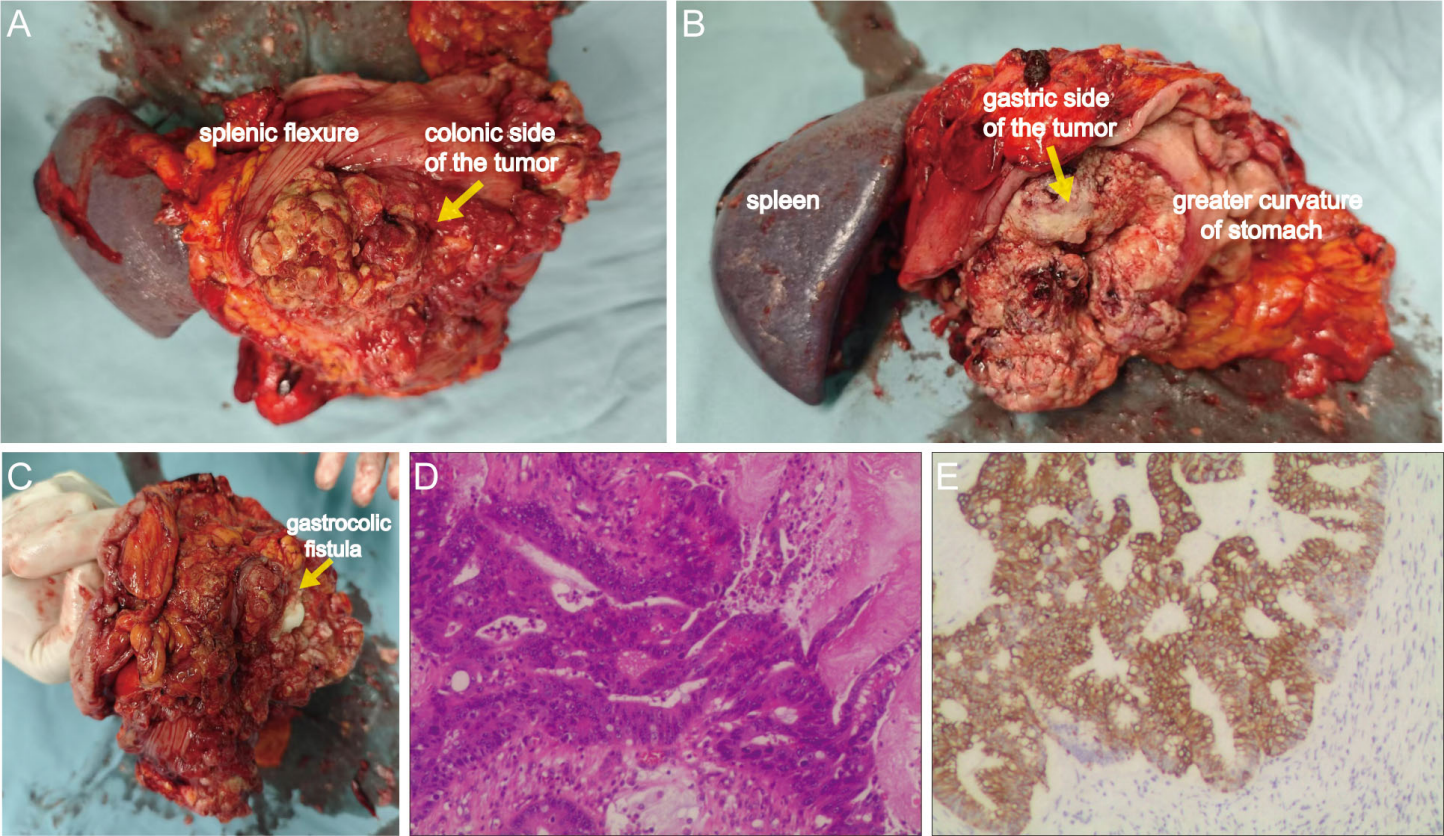
Gross surgical specimen and histopathological findings. **(A)** Anterior view of the tumor from the splenic flexure aspect. **(B)** Posterior view of the lesion from the gastric greater curvature aspect. **(C)** Identification of the gastrocolic fistula via exploration with fingers. **(D)** Hematoxylin and eosin (H&E) staining demonstrating moderately-poorly differentiated adenocarcinoma (100×). **(E)** Cytokeratin 20 (CK20) immunohistochemistry confirming colorectal origin (100×).

After adequate bowel preparation, en bloc resection was performed including left hemicolectomy, greater curvature gastrectomy (preserving >30% gastric volume), splenectomy with distal pancreatectomy, partial diaphragmatic resection. Intraoperative exploration confirmed a 13 ×12 ×10 cm tumor involving both the gastric body and the splenic flexure of the colon, with a gastrocolic fistula (**Figures 4A–C**). Histopathology confirmed moderately-poorly differentiated colonic adenocarcinoma (Stage IIIC pT4bN1bM0) with gastric wall penetration (**Figure 4D**). Intralymphatic cancer emboli were present while no neural invasion was identified. IHC demonstrated CK20+/CDX2+/SATB2+ (colonic phenotype) and CK7-/MUC5AC- (gastric marker negative) (**Figure 4E**). Three cycles of intraoperative hyperthermic intraperitoneal chemotherapy (HIPEC) with 5-fluorouracil (650 mg/m²) and oxaliplatin (200 mg/m²) were administered.

The patient received 6 cycles of adjuvant CapeOx chemotherapy postoperatively (capecitabine 1000 mg/m² twice daily days 1-14; oxaliplatin 130 mg/m² day 1 of each cycle, administered every 3 weeks). At the 12-month follow-up, no recurrence was observed.

## Discussion

Exophytic growth patterns represent rare manifestations in both gastric and colonic malignancies. While gastric cancer often exhibits extensive submucosal infiltration with a propensity for longitudinal spread along the gastric wall. In contrast, colorectal cancer predominantly demonstrates circumferential transmural infiltration leading to luminal narrowing. This case illustrates a diagnostically challenging scenario where exophytic transvisceral gastric invasion precipitated initial misdiagnosis. Notably, the splenic flexure adenocarcinoma demonstrated mixed endophytic-exophytic growth with exophytic predominance—an exceptionally rare manifestation of colon cancer. Previous reports, including two cases by Park et al. (2009) (5) and Wang et al. (2012) (6) of colon cancer masquerading as gastric stromal tumor with intact mucosa appearance, document similar diagnostic pitfalls. Comparatively, our case presented with more extensive gastric mucosal involvement, occult gastrocolic fistula and larger tumor dimensions, substantially increasing misdiagnosis risk. Because exophytic colon cancer is extremely rare, when the mass is large and adjacent to other organs, it is difficult to identify its primary site and misdiagnosis can easily occur. Hence, we performed a brief review of literature documenting cases where exophytic colonic carcinomas were misinterpreted as primary neoplasms of adjacent organs (**Table 1**).

**Table 1:** Summary of cases of exophytic colon cancer mimicking primary neoplasms of adjacent organs .

Author (year)	Country	Age, sex	Complaint	Tumor location	Misdiagnosis target	Size (cm)	Treatment (operative procedure)	Outcome
Okamoto (2005) (7)	Japan	74, F	Loss of weight, general fatigue, pain	Sigmoid colon	Ovarian cancer	4.0	Unknown	Unknown
Wang (2012) (6)	ROC	70, F	Dull epigastric pain, belching, and early satiety	Splenic flexure	GIST	5.0	Left hemicolectomy with lymph node dissection	Unknown
Park (2009) (5)	South Korea	62, M	dizziness	Splenic flexure	GIST	13.8×7.3	Wedge resection of the stomach, segmental resection of the transverse colon, distal pancreatectomy and splenectomy	Unknown
Munghate (2014) (8)	India	60, M	right sided pain abdomen, nausea, vomiting	Proximal transverse colon	Gallbladder cancer	4.0	Extended right hemicolectomy along with cholecystectomy	Unknown
Nair (2012) (13)	UK	76, M	Right upper quadrant pain, frequent Vomiting	Hepatic flexure	Gallbladder cacer/empyema	5.0	palliative gastro-jejunostomy and ileo-colic bypass	Unknown
Li (2017) (14)	PRC	39, M	Right low abdominal pain	Cecum and ascending colon	Retroperitoneal mesenchymoma	8.4×7.2	Right hemicolectomy and psoas major ablation	Unknown
Abdrabou (2014) (15)	Egypt	60, M	Abdominal swelling, vomiting and dyspepsia	ascending colon	Renal cell carcinoma	>10.0	Unknown	Unknown
The present (2024)	PRC	60, M	Melena and progressive fatigue	Splenic flexure	Gastric cancer	13×12×10	en bloc multivisceral resection including extended left hemicolectomy, partial gastrectomy, lymphadenectomy, distal pancreatectomy, splenectomy	12-month recurrence-free

M, male; F, female; GIST, Gastrointestinal Stromal Tumor.

Gastrocolic fistula, defined as abnormal tract connecting the colon and stomach, is a rare entity. Its pathogenesis encompasses both malignancies (e.g., gastric/colorectal carcinomas) and benign conditions (e.g., peptic ulcers or Crohn’s disease) (9, 10). In this case, an occult gastrocolic fistula was identified postoperatively. It is postulated that rapid tumor growth compressed and narrowed the fistulous tract, making it difficult to detect through endoscopy or CT examination. The fistula opening was prone to misinterpretation as a volcano-shaped gastric ulcer, creating diagnostic pitfalls. This mechanism also explains the absence of pathognomonic gastrocolic fistula symptoms—such as feculent vomiting or diarrhea—in the present case.

For GI malignancies with bleeding, a comprehensive preoperative assessment is imperative. Our experience underscores critical limitations of isolated upper GI assessment in gastrointestinal bleeding. Even with identified bleeding sources, complete lower GI evaluation remains imperative to exclude multifocal hemorrhage. Multidisciplinary consultation (MDT) becomes essential when diagnostic uncertainty persists. In the present case, preoperative assessment revealed an ill-defined tumor interface with the colon, necessitating imperative endoscopic evaluation. The two prior misdiagnoses by the previous hospital stemmed directly from the critical omission of colonoscopy. In the evaluation of malignancies demonstrating ill-defined margins or occult primary origin, integrated ^18^F-FDG PET/CT delivers essential diagnostic insights by mapping tumor metabolic activity. Current evidence supports its utility in primary tumor identification and detection of distant metastases, thereby optimizing staging accuracy and informing personalized treatment strategies. In this case, PET/CT verification of colonic involvement challenges the presumption of primary gastric malignancy, warranting distinguishment with colonic origins. In adittion, several diagnostic alternatives beyond PET-CT are available to prevent misdiagnosis. When initial CT reveals a large exophytic gastric mass, comprehensive immunohistochemical (IHC) analysis should be promptly performed on endoscopic biopsy specimens to determine gastrointestinal origin. If IHC and conventional endoscopy remain inconclusive, or more accurate staging is required, endoscopic ultrasound (EUS) or even diagnostic laparoscopy can be utilized to delineate the lesion’s origin and extent while obtaining histological confirmation. This case underscores the imperative for initiating an in-depth, multimodal diagnostic workflow when clinical manifestations contradict preliminary diagnoses, rather than proceeding directly to surgery.

Treatment strategies depend on primary tumor staging, surgical fitness, and metastatic burden. For locally advanced T4b lesions, en bloc multivisceral resection remains the cornerstone of curative management given the impossibility of distinguishing malignant from inflammatory adhesions intraoperatively (11). Our approach incorporated three evidence-based elements: First, organ-preserving major gastrectomy achieved R0 margins while preserving > 30% gastric volume, reducing surgical trauma and nutritional complications versus radical total gastrectomy. This tailored approach facilitated rapid postoperative recovery without complications. Second, prophylactic HIPEC addressed fistula-associated peritoneal recurrence risk, supported by PRODIGE-7 trial data demonstrating 43% recurrence reduction (12). Third, standardized adjuvant chemotherapy optimized systemic control. Collectively, these interventions balanced radical oncologic resection with functional preservation in this complex presentation.

This study has certain limitations that should be acknowledged. Firstly, the reported 12-month disease-free follow-up period, while encouraging, is insufficient to assess long-term oncological outcomes such as overall survival and late recurrence. This is particularly relevant given the aggressive nature of pT4b colonic adenocarcinoma, which often has a peak risk of recurrence beyond this timeframe. Secondly, as a single-case report from a single institution, our findings, though instructive, are influenced by the unique characteristics of the patient and the clinical setting, and thus may not be universally generalizable. Further prospective studies with larger cohorts and extended follow-up are warranted to validate the diagnostic and therapeutic approach described herein.

In summary, this case offers critical insights into trans-organ (stomach-colon) tumor invasion. Anatomically, the splenic flexure—anchored by the splenocolic ligament and shared gastroepiploic vasculature—establishes a direct pathway for gastric invasion. Furthermore, while exophytic gastric masses are primarily considered to be mesenchymal tumors (e.g., gastrointestinal stromal tumors, leiomyomas), malignancies originating from adjacent organs (including colon, pancreas, etc.) must be systematically excluded. For indeterminate exophytic gastric lesions, we advocate a comprehensive diagnostic evaluation encompassing endoscopy/endoscopic ultrasound-guided biopsy, PET-CT, and ultimately MDT consultation.

## Data Availability

The original contributions presented in the study are included in the article/supplementary material. Further inquiries can be directed to the corresponding author.
